# Non-attitudinal and non-knowledge based factors constrain households from translating good nutritional knowledge and attitude to achieve the WHO recommended minimum intake level for fruits and vegetables in a developing country setting: evidence from Gulu district, Uganda

**DOI:** 10.1186/s40795-021-00469-5

**Published:** 2021-11-09

**Authors:** Benjamin Kenyi Bendere Lomira, Prossy Nassanga, Daniel Micheal Okello, Duncan Ongeng

**Affiliations:** 1 Department of Food Science and Postharvest Technology, Faculty of Agriculture and Environment, Gulu University, Gulu, Uganda; 2 Department of Rural Development and Agribusines, Faculty of Agriculture and Environment, Gulu University, Gulu, Uganda

**Keywords:** Attitude, Knowledge, Fruits, Vegetables, Consumption

## Abstract

**Background:**

The high level of incidence of mortality attributed to non-communicable diseases such as cancer, diabetes and hypertension being experienced in developing countries requires concerted effort on investment in strategies that can reduce the risks of development of such diseases. Fruits and vegetables (FV) contain natural bioactive compounds, and if consumed at or above 400 g per day (RDMIL) as recommended by World Health Organization (WHO) is believed to contribute to reduced risk of development of such diseases. The objective of this study was to determine in a developing country set-up, the extent to which rural and urban households conform to RDMIL, the status of nutritional attitude (NA) and knowledge (NK) associated with consumption of FV, and to delineate non-attitudinal and non-knowledge-based factors (NANK) that hinder achievement of RDMIL.

**Method:**

A cross-sectional survey of 400 randomly selected households and 16 focus group discussions (FGD) were conducted using Gulu district of Uganda as a microcosm for a developing country setting. Level of consumption of FV was assessed using 24-h dietary recall and compared to RDMIL as a fraction (%). The status of NK and NA were determined using sets of closed-ended questions anchored on a three-point Likert scale. Further quantitative statistical analyses were conducted using t-test, chi-square, Pearson’s correlation and multiple linear regression. FGD provided data on NANK factors and were analysed using qualitative content analysis procedure.

**Results:**

Urban and rural inhabitants met up to 72.0 and 62.4% of the RMDIL, respectively, with absolute intake being higher among urban than rural households by 37.54 g. NK and NA were good but the intensity of NK was higher among urban respondents by 11%. RDMIL was positively correlated with NA while socio-demographic predictors of RDMIL varied with household location. FGD revealed that primary agricultural production constraints, market limitations, postharvest management limitations, health concerns, social discomfort and environmental policy restrictions were the major NANK factors that hindered achievement of the RDMIL.

**Conclusions:**

These results indicate that NANK factors constrain households from translating good NA and NK to achieve the RMDIL.

**Supplementary Information:**

The online version contains supplementary material available at 10.1186/s40795-021-00469-5.

## Background

The occurrence of nutrition-related health challenges of non-communicable diseases (NCDs) such as hypertension, cancer and diabetes have reached significant levels in developing countries [28]. Estimates over the last decades indicate that 34.5 million (65%) people died annually as a result of one or various combinations of NCDs. When segretated by age, of the 34.5 million deaths, over 14 million occurred among people within the age bracket of 16–69 years while 80% of them were found in developing regions of the world [33]. In those regions, and Africa in particular, statistics from selected countries including Uganda, Kenya, South Sudan, Rwanda, Benin, Mali, Ethiopia and Senegal indicate NCDs death incidence rates ranging from 24 to 43% [45]. However, considering the poor state of health services in many of the African countries, it is possible that some incidences are never recorded officially and therefore the reported statistics might actually be an underestimation.

Fruits and vegetables (FV) contain a considerable amount of natural dietary phytochemicals [40] which are believed to potentially contribute to reducing the chances of occurrence of NCDs [29]. Such bioactive compounds include alkaloids, saponins, flavonoids, isoflavonoids, tannins, terpenoids, polyphenols, anthocyaindins, phytoestrogens, glycosnoids, carotenoids, limonoids and phytoestrols [48]. The significance of fresh plant foods in management of NCDs is reflected in a previous report which indicates that frequent consumption of carotenoid-rich FV was associated with blood cholesterol level maintanence ostensibly through provision of antioxidants that reduce oxidative damage caused by low density lipoprotein oxidation. In addition, consumption of fruits including grapes, berries, apples and citrus was found to be effective in maintaining blood pressure because of their high contents of procyanidins, anthocyanins and flavonol compounds [42].

Relatedly, deficiency of essential micronutrients such as, vitamin A, iron and zinc is an important nutritional challenge largely experienced in developing regions of the world [16]. These regions are largely economically challenged and thus households in such localities are usually unable to access expensive but nutrient-rich animal source foods such as meat, fish and milk [20]. As a strategy to improve micronutrient deficiency in developing countries, sufficient intake of fresh FV has been recommended [49]. This is because FV are among the major sources of vitamins and minerals [55]. In consonant with the known significance of fresh FV consumption to human nutrition and health well-being, WHO [41] recommends daily consumption of a minimum (RDMIL) of 400 g (5 servings). Information on adherence to this recommendation at household and community level is important for public health planning.

A critical search of literature on this subject reveals that much of the information available on levels of consumption of fresh FV is largely available from developed countries [17] but very scanty for developing countries. However, due to the huge socio-economic differences that exist between developed and developing countries, information on consumption levels from developed countries can not be used realistically for public health planning in developing countries. In addition, considering the fact that developed countries such as those in Europe where intensity of nutrition education is high have so far achieved only up to 300 g per day [3], suggests that intake levels in developing countries might even be much lower. This situation calls for proper understanding of the factors that can explain lack of adherence to RDMIL. In the context of developing countries such as Uganda, such understanding should consider both the rural and urban set-up situations to provide opportunity for designing comprehensive strategies to foster adherence in both localities.

Previous food consumption related studies have revealed differences between rural and urban inhabitants with respect to consumption of industrially produced food products [46] and nutritional attitude (NA) was identified as a principal factor that influenced food choice but strongly moderated by nutritional knowledge (NK) [47]. However, with regard to fresh FV, limited information exists on how rural and urban inhabitants especially those in developing countries perceive the nutritional and health benefits associated with consumption of such plant foods, and the associated NK. In the context of this study, and paraphrasing the definition presented in UlHaq et al. [53], NA refers to “a learned predisposition to think, feel and act in a particular way with regard to consumption of FV”. Consumer attitudes regarding nutrition play a central role in developing preference and willingness to accept or reject particular food categories and is critical in making consumption choices [46]. NK is defined as the ability of individuals to acquire, process and understand nutrition information needed to make sound nutrition decisions [35]. Much of the information available on NA and NK with respect to consumption of various food categories has been derived from studies conducted among affluent societies in developed countries [38]. However, limited information on the same contexualized to developing country situations is available. In addition, it has been observed that NA and NK are affected by socio-demographic factors [50]. Considering that socio-demographic factors differ considerably between developed and developing countries [44] implies that information available from a developed country context can not easily be applied to a developing country context. Further more, in a developing country context, socio-demographic characteristics vary markedly between urban and rural set-ups. Therefore, to gain better understanding of how NA and NK affect consumption of FV it becomes paramount that data be gathered from both the urban and rural areas. Previous nutrition studies conducted in developing countries, especially in the domain of complementary feeding, have shown that good NA and NK may or may not translate into good nutrition practices [31, 37]. This suggests that certain non-attitudinal and or non-knowledge based factors could be at play. However, information on such factors and their influence on good nutrition practice such as adequate consumption of FV are largely lacking. Therefore, the objective of this study was to determine, in a developing country context, the extent to which rural and urban households conform to RDMIL, the status of NA and NK associated with consumption, and non-attitudinal and non-knowledge-based (NANK) factors that hinder achievement of RDMIL.

## Methods

### Study area and study population

**Fig. 1:**
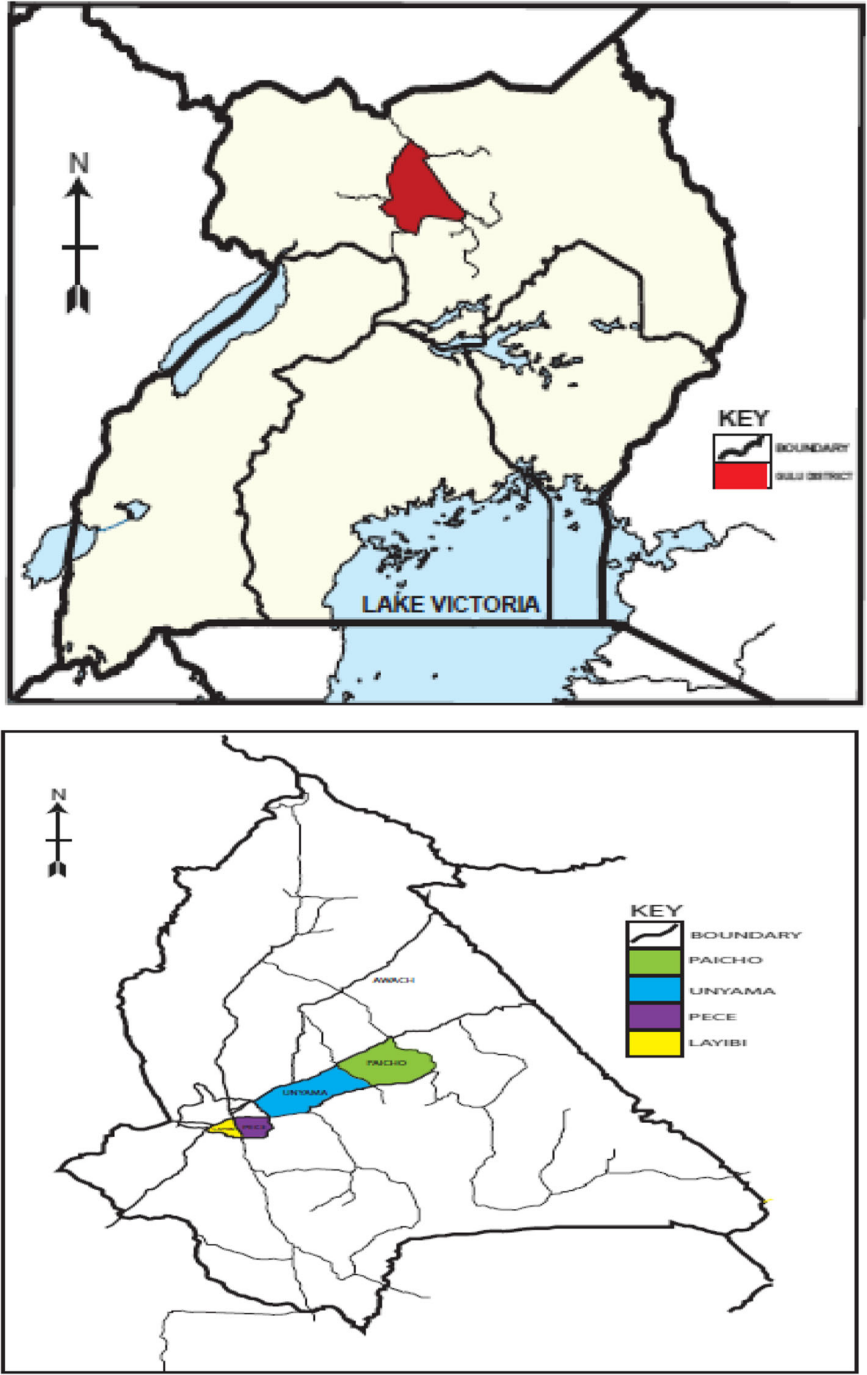
Map of the study area. Map is an original production by the authors

The study was conducted in Gulu district which is located in Northern Uganda between longitude 30^0^ -32^0^ East and latitude 2^0^–4^0^ North. It is bordered by Amuru district from the West, Pader district from the East, Lamwo district from the North East and Omoro district from the South. The total land area of the district is 3.449.08 km^2^ which is 1.44% of the land size of Uganda [23]. The population of Gulu district according to the 2014 census was projected to be 443,733 people [52]. The district experiences a climatic regime characterized by dry and wet seasons, with average annual rainfall of 1500 mm/ annum. It also experiences a monthly average rainfall variation of 1.4–230 mm between January and August, respectively [23]. The study population comprised of households that were officially registered by local authorities in the district. The main inclusion criteria were that the household had been resident in the district for at least 6 months and the person in charge of food preparation in the household was willing to participate in the study. Map of the study area is presented in Fig. 1.

### Study design, sample size and sampling framework

A cross-sectional study design that made use of survey questionnaires for individual household interviews and a guide for focus group discussions was applied. The survey questionnaire was used to collect quantitative data while the focus group discussion guide was used to collect qualitative data. The sample size, defined in the context of this study as the number of households (n) that participated in the study was calculated using a standard formula according to Israel [26].

1 $$ n=\frac{N}{1+N{(e)}^2} $$

Where, n is the sample size, N is the population size of Gulu district (443,73 3[52]), *e* is the marginal error level fixed at 0.05. On the basis of Eq. 1 and parameter values already defined, the sample size (n) for individual household interview was determined to be 400 households. This being a comparative study, the calculated sample size was divided into two resulting into 200 households each for rural and urban setting, respectively.

Following the determination of the sample size, a multi-stage sampling procedure was used to locate the participating households. First, two sub-counties and two divisions were randomly selected from the rural and urban areas of the district, respectively. From these, two parishes were selected randomly from each sub-county for the rural setting and two parishes from each division for the urban setting. This resulted into a total of 8 parishes. In stage three (3), two villages were randomly selected from each of the parish resulting into a total of 16 villages (8 from the rural area and 8 from the urban area). In stage four (4), 25 households were selected randomly from each village, resulting into a total of 400 households (200 from rural area and 200 from the urban area) consistent with the sample size determined according to Eq. 1. The respondent for individual household interview was the member of the household in charge of food preparation.

For FGD sessions, in order to ensure originality of information, households selected to participate in in-depth interviews were not selected to participate in FGDs. For each location (urban, rural), eight (8) FGDs were conducted with each consisting of 8–10 individuals. The eight FGDs used in each location (total = 16) is above the minimum number of six required for saturation of information in qualitative studies [51]. As was the case for the individual household interview, participants for the FGDs were also household members in charge of food preparation.

### Study instruments

Data on consumption of FV among households were collected using a 24-h dietary recall tool and procedure previously used by Hongu et al. [24] and Salehi et al. [47]. The tool was modified to reflect only those FV that are consumed in the study area. NA and NK were assessed using a standard questionnaire adapted with modification from FAO [15]. The modification was made to reflect only issues related to consumption of FV. The NK section had 16 closed-end questions framed (negatively or positively) to test knowledge about the importance of FV to nutrition and health well-being. The questions were anchored on a 3-point likert scale (1 = agree, 2 = neither agree nor disagree and 0 = disagree). The NA section had 28 closed-ended questions designed to test respondents’ attitude towards consumption of FV. As was the case for NK, questions for NA were also anchored on a 3-point Likert scale (0 = disagree, 2 = neither agree nor disagree, 1 = agree) according to Anand and Puri [1]. The questionnaire also had a provision for collection of data on socio-demographic characteristics of the households (Supplementary material S1).

For the FGD, a guide adapted with modification from Duthie et al. [12] and Salehi et al. [47] was used. The guide was modified to generate information on NANK factors that hinder achievement of RDMIL among households (Supplementary material S2). The instruments were pretested among selected households in non-participating villages in the study area. This was done to ensure accuracy, clarity and consistency in the interpretation of the questions. After pretesting, responses were analyzed to check for validity and ambiguous questions were rephrased. Questions used to test NA and NK, were subjected to the Cronbach test. The test resulted in a reliability index of 0.72 and 0.77 for NA and NK, respectively. These levels of reliability are considered acceptable in nutrition research [22].

### Data collection

Data was collected using research assistants who had previous exposure to nutrition surveys and fluent in both English and local language of the study area (Luo/Acholi). In order to ensure that the assistants did not interfere with originality of the information, they were further trained on how to inteprete questions to the study participants but not to assist them in providing answers. Data was collected in stages. First stage was assessment of daily consumption levels of FV. Here, each respondent (the person in the household responsible for food preparation) was asked to mention the FV that were consumed by household members in the past 24 h prior to the study. However, to provide a fair estimate of the quantities consumed, representative portions of the food items consumed were weighed and recorded in a standard unit (grams) commonly used in nutrition studies and assesments. The persons in charge of food preparation were interviewed from their respective homes. The second stage was assessment of NA and NK. The third stage was assessment of socio-demographic characteristics of the respondents. The fourth stage was the FGD sessions for which each session lasted 1.5–2 h.

### Data analysis

Data on socio-demographic characteristics were summarized using descriptive statistics (frequency, percentages, mean and standard deviation where applicable). Chi square test was perfomed to compare categorical socio-demographic variables among rural and urban respondents. To determine the extent to which households conform to RDMIL, the combined amount (g) of FV for each household (derived from the 24-h recall), adjusted for the number of household members in adult equivalent, were summed up and divided by the number of households that participated in the study. The average value for rural or urban area was compared with the RDMIL as a fraction (%). Independent 2-sample t-test was used to compare mean consumption of each of the FV, overall mean for fruits or vegetables, and the combined overall mean for FV between rural and urban households following ascertainment of conformity to normality requirement according to Kolmogorov-Smirnov test.

With regard to NK, the analysis went through several steps. On the basis of the answers to questions on knowledge elements provided by the respondents, every correct answer was given a score of one (1) while a wrong answer and where the respondent did not know was awarded a score of zero (0). Scores for each respondent were calculated by summing up the scores attained for each question to generate the total NK score which ranged between zero and sixteen (0–16). The overall score was ranked as good or poor depending on the score level. A rank of “good” was given if the overall score stood at 55% and above or poor if it fell below 55% as previously reported by Ul Haq et al. [53]. With regard to NA, before analysis, responses were also scored as in the case of NK. Therefore, a score of zero (0) was awarded for disagree, two (2) for neither agree nor disagree and one (1) for agree. Reverse scoring was done for negatively-framed statements. This means that a score of 0 was given to ‘agree’ and 1 to ‘disagree’ [1]. Scores for each respondent were calculated by summing up the scores attained from each question and the overall score ranked as good or poor. The NA was ranked as good for overall scores of 57.1% and above. Otherwise the human factor was ranked as bad [53]. Independent 2-sample *t*-test was also used to compare the mean difference in NK or NA score between urban and rural respondents following ascertainment of conformity to normality requirement according to Kolmogorov-Smirnov test. Bivariate analysis (Pearson’s correlation) was used to determine the association between NA, NK and RDMIL. On the other hand, multiple linear regression was performed to establish socio-demographic predictors of the level of consumption of FV. Before running the regression, a number of diagnostic tests were conducted. The Shapiro-Wilk test for nornmality was conducted on the dependent variable (level of consumption of FV) and continuous independent variables (household size, age and education level of the respondent). The results showed that the dependent variable was normally distributed (*p* value = 0.060) while the continuous independendent variabes were not (*p* < 0.05). Therefore, the continuous independent variables were transformed to natural logarithm and conformed to the normality assumption on the basis of the histogram. Pearson’s correlation analysis was run in order to eliminate highly correlated independent variables with a correlation coefficient greater than 0.70 [11]. Following the diagnostic tests, three models were estimated. Model 1 was for the pooled dataset (urban and rural locations combined), with the locational indicator included as an independent variable while models 2 and 3 were location specific. Model 2 was for rural dataset and model 3 was for the urban dataset. The purpose of models 2 and 3 were to show whether the two locations have the same factors influencing level of consumption of FV. The general model depicting the variables used is presented in Eq. 2.

2 $$ {Y}_i=\alpha +{\beta}_1{X}_1+{\beta}_2{X}_2+{\beta}_3{X}_3\dots \dots \dots \dots \dots \dots \dots {\beta}_n{X}_n+\mu $$

From Eq. 2, Y_i_ is the level of consumption of FV, α is the regression constant; β is the regression coefficient, X_1_ to X_n_ are the independent variables and μ is the error term. The following independent variables were selected and used to run the regressions:location (1 = rural, 0 = urban), age of the respondent (years), sex of the respondent (1 = male, 0 = female), marital status of the respondent (1 = married, 0 = otherwise), major occupation of the household head (1 = farm-based, 0 = non-farm based), education level of the respondent (years), attendance of health education by the respondent (1 = yes, 0 = no), household size (number), respondent’s attendance of health education (1 = yes,0 = no), main source of household income (1 = farming, 0 = non-farm activities), woman decides on how family income is used (1 = yes,0 = no), woman decides on the type of food eaten at home (1 = yes, 0 = no). After running the regressions, three post-estimation tests were conducted. First, the Ramsey Regression Specification Error (RESET) test for linear model specification was conducted and revealed that selection of the linear model was appropriate (F = 0.46, *p* = 0.7074). Secondly, the Breusch-Pagan/Cook-Weisberg test for heteroskedasticity was conducted and showed no evidence of heteroskedasticity (^χ2^ = 0.15, *p* value = 0.6965). Lastly, the variance inflation factor (VIF) test for multi-collinieraity showed that the independent variables had mean VIF of 1.26 and a maximum value of 1.50 indicating no problem of multi-colinieraity in the regression models. Regression analyses were performed using STATA version 14 while other stastistical analyses were performed using Statistical Package for Social Sciences (SPSS) version 2, and the level of significance was fixed at 5%. However, for tests on data comparing categorical socio-demographic variables, consumption of FV between rural and urban households, a Bonferroni correction test was conducted resulting into a corrected significance level of 0.004 and 0.002, respectively. Finally, in order to establish NANK factors that limit consumption of FV data from FGDs were summarized using qualitative content analysis as described by Elo & Kyngäs [13]. This was achieved by determining the units of analysis followed by categorization of the data drawing inferences on the basis of the different categories.

## Results

### Socio-demographic characteristics of the study households

Results of Chi-square test for socio-demographic characteristics and location of residence of study participants are presented in Table 1.

**Table 1:** Chi-square test for socio-demographic characteristics and location of study respondents

Socio- demographic variables	Location of residence					
	Rural (***n*** = 200)		Urban (***n*** = 200)		X^**2**^	***P*** value*
	N	%	N	%		
**Sex of the respondent**					0.296	0.586
Male	8.0	4.0	6.0	3.0		
Female	192	96	194	97.0		
**Marital status of the respondent**					27.615	**0.000**
Single	18	9.0	30	15.0		
Married	122	61.0	146	73.0		
Separated	19	9.5	16	8.0		
Widowed	41	20.5	8	4.0		
**Occupation of household head**					25.570	0.019
Not Employed	40	20.0	34	17.0		
Employed (Salaried)	18	9.0	27	13.5		
Small Scale Trading	46	23.0	81	40.5		
Casual Labor	7	3.5	18	9.0		
Farming	84	42.0	21	10.5		
Others	5	2.5	19	9.5		
**Attendance of school by the respondent**					3.735	0.053
No	22	11.0	16.0	8.0		
Yes	178	89.0	184	92.0		
**Education level of the respondent**					32.581	0.013
No formal education	15	7.5	9.0	4.5		
Primary	113	56.5	65	32.5		
Secondary	57	28.5	108	54.0		
Post secondary	14	7.5	18	9.0		
**Attendance of health education by the respondent**					0.795	0.375
No	57	28.5	37	18.5		
Yes	143	71.5	163	81.5		
**Number of times the respondent attended health education**					5.553	0.135
Never	57	28.5	37	18.5		
Once	13	6.5	23	11.5		
Twice	17	8.5	26	13.0		
More than twice	113	56.5	114	57.0		
**Attendance ofnutrition education by the respondent**					0.015	0.902
No	75	37.5	63	31.5		
Yes	125	62.5	137	68.5		
**Frequency of attendance of nutrition education by the respondent**					10.974	0.012
Never	75	37.5	63	31.5		
Once	14	7.0	13	6.5		
Twice	23	11.5	20	10.0		
More than twice	88	44.0	104	52.0		
**Main source of household income**					48.871	**0.000**
Formal employment	22	11.0	29	14.5		
Casual labor	15	7.5	19	9.5		
Small scale business	70	35.0	105	52.5		
Sale of agricultural produce	86	43.0	30	15.0		
Farming	5.0	2.5	3.0	1.5		
Others	2.0	1.0	14	7.0		
**How food is obtained in the household**					149.862	**0.000**
Farming	152	76.0	32	16		
Purchase	46	23.0	165	82.5		
Food aid	1.0	0.5	1.0	0.5		
Transfers from friends	1.0	0.5	2.0	1.0		
**Responsibility of providing food for the household**					41.256	**0.000**
Father	44	22	78	39		
Mother	97	48.5	42	21		
Both father and mother	43	21.5	70	35		
Relatives	16	8.0	10	5.0		
**Decision on the type of food to eat in the household**					8.483	0.132
Husband	9.0	4.5	7.0	3.5		
Wife	155	77.5	148	74		
Both wife and husband	22	11	34	17		
Children	2	1.0	6.0	3.0		
Grandmother	12	6.0	5.0	2.5		
**Decision on how family income is used**					33.669	**0.000**
Husband	27	13.5	59	29.5		
Wife	84	42	39	19.5		
Both wife and husband	77	38.5	96	48		
Grandmother	12	6.0	6.0	3.0		

^*^Statistical significance is based on Bonferroni correction value of 0.004

Generally, significant association in socio-demographic characteristics among rural and urban respondents were observed with respect to marital status, main source of family income, how food is obtained in the household, responsibility of providing food for the household and decision on how family income is used. Out of the total study participants, 61% of them from rural areas were married compared to 73% who were from the urban areas. The main source of family income for rural households was sale of agricultural produce (43%) while for urban residents was small-scale business (52.5%). When asked about how food was obtained in households, the largest proportion of respondents from rural areas (76%) reported that food was majorly obtained through own production (farming) while majority of their urban counterparts (82.5%) obtained theirs through purchase from the market. Relatedly, the responsibility of providing food for the household was majorly (48.5%) in the hands of women for the case of rural households while in the urban areas the responsibility was handled by male household head (39%). With regard to the decision on how family income is spent, in most of the rural households, the decision was mainly taken by wives (42%) unlike in the urban households were the decision was majorly (48%) a consensus between the wife and the husband. The mean age of the respondents was 36.6 ± 14.6 (minimum: 18; maximum: 70) and 29.8 ± 9 (minimum: 18; maximum: 64) for rural and urban households, respectively.

### The status of consumption of fruits and vegetables

Data on the mean daily consumption of individual FV among rural and urban households are presented in Table 2. Results of an independent sample t-test revealed no significant difference in combined mean daily consumption of vegetables between rural and urban households. However, with regard to fruits, significant difference was observed between rural and urban households. Specifically, consumption of fruits was higher among urban than rural inhabitants by about 84.93 g.

**Table 2:** Mean daily consumption of various fruits and vegetables segregated by location of residence

	Consumption level (grams)			
	Rural (***n*** = 200)	Urban (***n*** = 200)		
Fruits/Vegetable species	Mean % ± SD	Mean % ± SD	Mean difference	*P*-value*
**Vegetables (V)**				
*Brassica capitate* (Cabbage)	46.7 ± 4.7	14.5 ± 2.65	32.13	**0.000**
*Solanum lycopersicum* (Tomatoes)	24 ± 1.6	37.2 ± 2.4	−13.3	0.042
*Spinacia oleracea* (Spinach)	3 ± 1.5	0.93 ± 0.81	2.1	0.015
*Brassica botrytis* (Cauliflower)	0.6 ± 0.6	0.8 ± 0.8	−0.12	0.688
*Capsicum annuum* (Sweet pepper)	0.8 ± 0.3	15 ± 0.6	−0.7	0.020
*Daucuscarota* (Carrot)	0.63 ± 0.34	4.1 ± 1.1	3.5	**0.000**
*Amaranthus retroflexus* (Amaranthus)	25.8 ± 3.5	21.3 ± 3.4	4.5	0.214
*Solanum tuberosum* (Potatoes)	14.93 ± 2.6	3.2 ± 1.14	11.7	**0.000**
*Vigna unguiculatus* (boo)	31.9 ± 3.5	20.1 ± 2.95	11.7	**0.000**
*Hibiscus spp* (malakuang)	20.7 ± 3.4	11.5 ± 2.5	9.2	**0.000**
*Solanum melongena* (Eggplant)	24.5 ± 3.61	7.8 ± 1.96	16.72	**0.000**
*Solanum gilo* (Tula)	5.6 ± 1.8	10.3 ± 2.3	−4.7	**0.001**
*Phaseolus vulgaris* (Fresh bean)	25.7 ± 3.7	5.8 ± 1.6	−19.9	**0.000**
**Total consumption for V**	**227.98 ± 8.43**	**219.26 ± 7.72**	−8.72	0.067
**Fruits (F)**				
*Ananas comosus* (Pineapple)	2.3 ± 0.91	7.2 ± 1.71	−4.9	**0.000**
*Citrullus lanatus* (Watermelon)	0.8 ± 0.6	6.8 ± 1.7	−5.97	**0.000**
*Musa spp* (Sweet banana)	4.3 ± 1.21	15 ± 2.3	−10.7	**0.000**
*Persea americana* (Avocado)	8.6 ± 1.64	8.2 ± 1.6	−0.4	0.766
*Citrus spp* (Oranges)	8.8 ± 1.81	8 ± 1.7	0.8	0.526
*Malus* (Apple)	1.990 ± 0.9	3.4 ± 1.4	−1.4	0.087
*Mangifera indica* (Mangoes)	20.9 ± 2.62	28.9 ± 2.93	−7.96	**0.000**
*Artocarpus heterophyllus* (Jackfruit)	6.7 ± 1.5	5.8 ± 1.54	−0.1	0.388
*Passiflora edulis* (passion fruit)	4.8 ± 1.3	5.6 ± 1.63	−2.8	0.006
Fruit juice	0.000 ± 0.000	23.5 ± 2.7	−23.5	**0.000**
**Total consumption for F**	**59.1 ± 3.99**	**144.03 ± 4.7**	**84.93**	**0.001**
**Total consumption for FV**	**249.51 ± 6.2**	**287.05 ± 6.4**	**37.54**	**0.001**

^*^Statistical significance is based on Bonferroni correction value of 0.002

With respect to consumption of individual FV investigated, significant variations were observed between rural and urban respondents. Amongst vegetables investigated, significant difference was observed in the consumption of *Brassica capitate*, *Daucus carota*, *Solanum tuberosum*, *Vigna unguiculatus*, *Hibiscus spp.*, *Solanum gilo*, *Solanum melongena* and *Phaseolus vulgaris-*fresh form. Specifically, urban households had higher mean daily consumption of *Daucus carota* and *Solanum gilo* while rural households had higher mean daily consumption of *Brassica capitate*, *Vigna unguiculatus*, *Hibiscus spp*., *Solanum tuberosum* and *Phaseolus vulgaris* (fresh bean). With regard to fruits, significant mean differences were observed for *Ananas comosus*, *Citrullus lanatus*, Musa *spp., Mangifera indica* and fresh fruit juice. Across all the fruits, urban households had higher consumption scores compared to their rural counterparts. Irrespective of the location, the observed total daily consumption levels for FV was below the RMDIL. Specifically, urban and rural households were only able to meet the RMDIL by 72 and 62.4%, respectively.

### Socio-demographic predictors of consumption of fruits and vegetables

Results of multiple linear regression on socio-demographic factors that can predict consumption of FV among households are presented in Table 3. The locational indicator (urban, rural) was significant suggesting that the level of consumption of FV differred between urban and rural households. The standardized regression coeffient of the locational indicator predicted that consumption of FV was significantly lower among rural households than among urban counterparts by 23%. In the pooled regression model, the main source of household income, age of the respondent, education level of the respondent, and attendance of nutrition training by the respondent positively predicted consumption, while, martial status of the respondent and household size negatively predicted consumption of FV instead. In the case of negative prediction, two scenarios were apparent. First, an increase in household size was associated with 13% reduction in consumption of FV while a state of the respondent being married was associated with lower consumption of FV by 11%. In the case of positive prediction, the following scenarios sufficed. First, a situation of farming being the main source of household income was associated with 17% higher consumption of FV. Secondly, attendance of nutrition training by the respondent was associated with 19% higher consumption of FV. Thirdly, an increase in age of the respondent was associated with a 13% increase in consumption of FV. Lastly, an increase in the level of education of the respondent was associated with 28% increase in consumption of FV. When data was segregated by location, regression models 2 and 3 showed that only the level of education of the respondent was a significant positive predictor among both rural and urban households. In quantitative terms, an increase in the level of education of the respondent was associated with 27 and 28% increase in consumption of FV among rural and urban households, respectively. However, matital status of the respondent, main occupation of the household head, attendance of health education, attendance of nutrition training by the respondent, and main source of household income were the significant predictors in the rural area (model 2) while only age of respondent and household size were significant in the urban area (model 3). In terms of the magnitude of prediction, for model 2 (depicting the rural area), the following scenarios were apparent. First, respondent being married was associated with 14% lower consumption of FV. Secondly, farming as a main occupation of the household head was associated with 20% lower consumption. Thirdly, attendance of health education by the respondent was associated with 19% lower consumption. Fourthly, attendance of nutrition training by the respondent was associated with a 30% higher consumption. Fifth, farming as the main source of household income was associated with 20% higher consumption of FV. On the other hand, in terms of the magnitude of the prediction for model 3 (urban area), the following scenarios were apparent. First, an increase in the age of the respondent was associated with 22% increase in level of consumption while an increase in household size was associated with 21% reduction in consumption of FV. Further more, results show that main occupation of the household head and attendance of nutrition training were not significant factors in the pooled model (model 1) neither in the model for the urban area (model 3) but were peculiar in the model for the rural area (model 2).

**Table 3:** Socio-demographic predictors of consumption of fruits and vegetables segregated by location of residence

	Predictor parameters								
Socio-demographic factors	Pooled (*n* = 400)			Rural (*n* = 200)			Urban (*n* = 200)		
	Β	S.E	*P*-value	Β	S.E	*P*-value	Β	S.E	*P*-value
Location of the respondent (1 = rural, 0 = urban)	−0.232	9.041	**0.000**						
Sex of the respondent (1 = male, 0 = female),	0.015	24.624	0.756	0.019	31.204	0.787	0.057	39.758	0.447
Age of the respondent (log transformed) (years)	0.135	13.045	**0.008**	−0.072	16.136	0.331	0.215	23.317	**0.003**
Marital status of the respondent (1 = married, 0 = otherwise)	−0.115	10.675	**0.039**	−0.138	14.117	**0.028**	−0.131	16.393	0.105
Major occupation of the household head (1 = farm-based, 0 = non-farm based)	−0.086	10.659	0.076	−0.195	12.539	**0.004**	0.089	19.560	0.237
Education level of the respondent (log transformed) (years)	0.280	6.830	**0.000**	0.268	9.240	**0.001**	0.280	10.260	**0.000**
Attendance of health education by the respondent (1 = yes, 0 = no)	−0.086	8.995	0.079	−0.188	12.222	**0.007**	0.020	13.519	0.782
Attendanceof nutrition training (1 = Yes,0 = no)	0.193	10.922	**0.000**	0.298	14.645	**0.000**	0.107	16.182	0.140
Household size (log transformed) (numbers)	−0.135	6.741	**0.004**	−0.094	8.613	0.149	−0.206	10.816	**0.003**
Main source of household income (1 = farm-based, 0 = non-farm based)	0.168	9.802	**0.001**	0.197	11.701	**0.004**	0.070	18.074	0.346
Woman decides on how family income is used (1 = Yes, 0 = No)	−0.081	11.329	0.158	−0.074	14.712	0.376	−0.097	17.538	0.206
Woman decides on type of food eaten in the household (1 = Yes, 0 = others)	−0.039	10.483	0.435	−0.049	15.1780	0.499	−0.064	14.645	0.369
Constant	107.189	58.879	0.0069	176.120	72.725	0.016	−55.598	98.420	0.573
F value	9.47			6.41			3.96		
*P* > f	0.0000			0.0000			0.0000		
R-squared	0.2265			0.2729			0.1874		
Adjusted R-squared	0.2026			0.2304			0.1401		

*B* Stadardized coefficient, *SE* Standard error

### The status of nutritional attitude associated with consumption of fresh fruits and vegetables

**Fig. 2:**
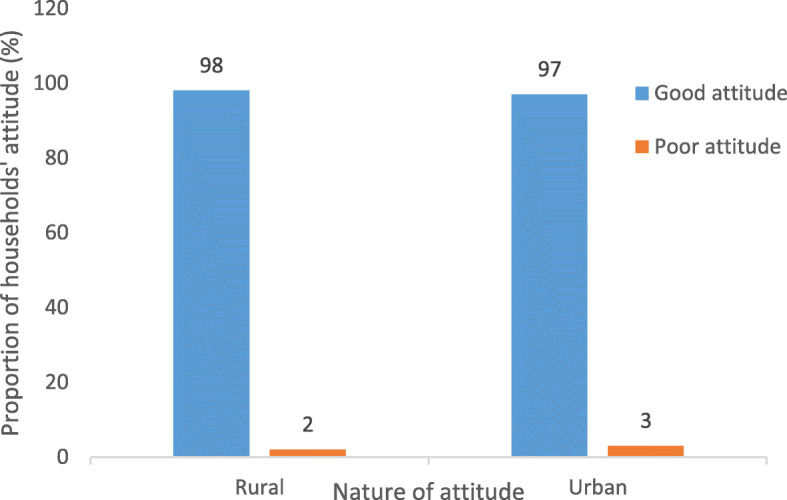
Distribution of respondents’ attitude towards consumption of fruits and vegetables segregated by location of residence

The status of NA associated with consumption of FV among rural and urban households is presented in Fig. 2.

In general, majority of the respondents from both rural and urban areas had good NA towards consumption of FV. Application of independent sample t-test revealed no significant difference in the status of NA among inhabitants residing in the two locations investigated (Rural: *n* = 200, Mean ± SD = 98 ± 0.14; Urban: *n* = 200, Mean ± SD = 97 ± 0.17; *p* = 0.523).

### Status of nutritional knowledge associated with consumption of fresh fruits and vegetables

**Fig. 3:**
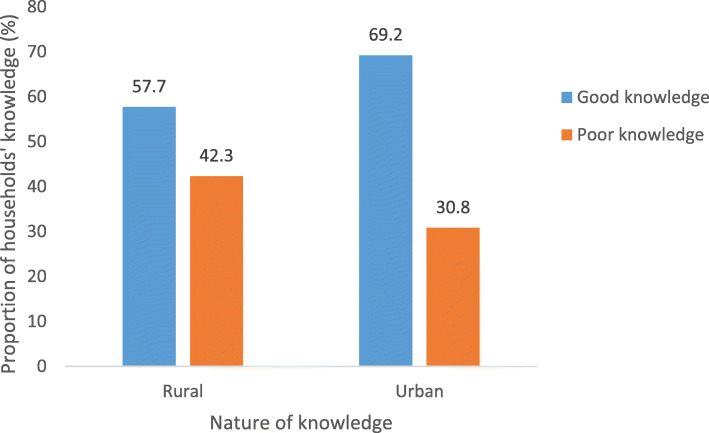
Distribution of the status of knowledge associated with consumption of fruits and vegetables segragated by location of residence

The status of NK associated with consumption of FV among rural and urban respondents is presented in Fig. 3.

Generally, irrespective of the location of residence, the proportion of respondents that exhibited good NK was more than 50%. Nonetheless, a higher proportion of urban respondents were knowledgeable than their rural counterparts by a difference of about 11% (Rural: *n* = 200, Mean ± SD =58 ± 0.495; Urban: *n* = 200, Mean ± SD = 69 ± 0.463; *p* = 0.017).

### Association between nutritional attitude, nutritional knowledge and consumption of fruits and vegetables

Results of bivariate analysis (Pearson’s correlation) are presented in Table 4. The results revealed a positive and significant relationship between NA and FV consumption among both rural and urban households. However, no association was observed between NK and FV consumption irrespective of the location.

**Table 4:** Association between nutritional attitude, nutritional knowledge and consumption of fruits and vegetables

	Rural (*n* = 200)			Urban (*n* = 200)		
	NA	NK	FV	NA	NK	FV
NA	1			1		
NK	−0.087	1		0.018	1	
FV	0.164^a^	−0.009	1	0.141^a^	0.040	1

Values are correlation coefficients

^a^Correlation is significant at 5% (2-tailed)

### Non-attitudinal and non-knowledge-based factors that hinder consumption of fruits and vegetables

Information on NANK factors that hindered consumption of FV, generated during FGDs segregated by respondents’ location of residence is presented in Table 5. In general, both rural and urban respondents experienced similar NANK barriers. However, majority of the barriers (73.7%) were experienced in the rural areas.

**Table 5:** Non- attitudinal and non- knowledge-based factors that hinder consumption of fruits and vegetables

Factor	Description	Respondents’ location	
		Rural	Urban
Limited budget	Households are unable to purchaseexpensivelyFV from the market during off-season due to lack of financial resources	✓	✓
Pest and diseases	Pests and disease pressure is too high and impacts heavily on the yield of FV because the existing varieties are not resistant	✓	✓
Unpalatability	Some vegetables have unpleasant taste hence they are not liked by children and teenagers	✓	✓
Allergic disorders	Some FV cause allergic disorders to some people so they can not be consumed	✓	**X**
Distance to markets	Remote markets discourage households from accessing FV due to long distances	✓	✓
Seasonality	Some FV are not available throughout the year especially during the dry season	✓	✓
Unfavourable environmental policies	Existance of policies that prevent utilization of wet land in dry season hinder vegetable production during this period of the year	✓	**X**
Disturbance by animals	Unrestricted movement of animals especially during the dry season leads to destruction of vegetable crops	✓	**X**
Unvailability	Certain FV are unavailable in the nearby markets even during on season	✓	**X**
Occurrence of hailstorms	Destruction that occurs due to hail storms action results into reduced amount of FV produced	✓	**X**
Stigma	The notion that regular consumption of FV is associated with poverty makes households try as much to reduce their consumption	✓	✓
Land allocation prioritization	Most land is allocated to cash crops such as soybean andmaize at the expense of FV	✓	✓
Lack of preservation technologies	Preservation of fresh FV hardly takes place due to lack of cold chain technologies and high electricity costs	**X**	✓
Bad weather	Prolonged dry spells lead to reduction in quantity of FV. In addition, heavy rains destroy crops. Heavy rais are also associated with high incidences of FV diseases	**X**	✓
Medical conditions	Presence of disease conditions, e.g., ulcers that prevent some people from consuming certain FV	**X**	✓
Environmental limitations	Peculiar environmental requirements can not allow production of certain types of FV	**X**	✓

## Discussion

The health and nutritional significance associated with consumption of FV justifies the need for investment in efforts to enable households consume quanties required to effect positive nutritional and health effects. The results have demonstrated that households in both rural and urban localities were unable to meet the RDMIL although intake by urban households was better than that of their rural counterparts. This implies that generally both localities require efforts in order to improve consumption, but specifically more efforts should be dedicated to rural areas. When consumption was segregated into FV, the combined mean daily consumption level of fruits was significantly higher among urban than rural households while that of vegetables was identical between the two localities. This implies that the higher consumption level of FV reported among urban than rural households may be due to differentials in the itake of fruits. Another dichotomy observed between rural and urban households was interms of the types of FV consumed. In the case of vegetables, tomatoes, cauliflower, sweet pepper, carrot, and tula were largely consumed by urban residents as opposed to rural dwellers while cabbage, spinach, amaranthus, potatoes, boo, malakuang, egg plant, and fresh beans were more consumed in rural than in urban areas. On the other hand, for fruits, pineapple, watermelon, sweet banana, apple, mangoes, passion fruits and fruit juice were largely consumed by urban dwellers than the rural inhabitants. These observations illustrate that nutritious and expensive FV are largely consumed by urban dwellers while cheaper types are consumed by rural households. This is likely due to better socio-economic situation among urban compared to rural inhabitants.

Results of regression analysis revealed that overall, location was a significant factor that predicted consumption of FV in the study area. The results of the pooled model indicating higher consumption of FV among urban compared to rural households by 23% corroborates with the outcome of the independent sample t-test which revealed that absolute daily consumption of FV was higher among urban than rural households by 37.54 g. This implies that location of the household is an important factor that should be taken into condideration in designing interventions to improve consumption of FV in localities such as Gulu district. The effect of location was also reflected in the pattern of the significance of various socio-demographic factors that predicted consumption of FV. This is because, whereas, age, marital status, education level of the respondent, attendance of nutrition training by the respondent, houseold size and the main souce of household income were significant in the pooled model (model 1), only marital status, attendance of nutrition training by the respondent and the main source of household income were significant among rural households (model 2) while age of the respondent and household size were significant among urban households (model 3) instead. This implies that location does not only influence level of consumption but also provides indications on other location-specific factors that may influence the level of consumption of FV as well. A typical scenario is the fact that the main occupation of the household head and attendance of health education by the respondent were only significant in the rural area (model 2) but not in the urban area (model 3) neither in the pooled model (Model 1). The observed location-specific nature of socio-demographic predictors is not peculiar to this study. It has also been reported for daily FV intake among 11-year old school children in Europe (De [9]) and dietary diversity among children 6–23 months in Benin [36]. An important observation revealed by the regression analysis is the fact that education level of the respondent was the only significant factor that cut accross both locations and was positive in nature. This may not be surprising because level of education is a universal factor that has been reported to positively influence consumption of FV in other localities such as the United States of America [2].

Consumer attitude towards food consumption has been variously illustrated to depend on food type ([10],) but strongly modulated by socio-cultural and socio-demographic factors [43]. In the context of this study, due to marked differences in the socio-demographic variables observed between urban and rural households, it was anticipated that NA of rural and urban respondents would also vary. To the contrary, NA was identically good among respondents from the two localities. NK is generally believed to be one of the key factors that moderate the evaluative effect of attitude on food choice [27]. Based on the results of the current study, it is apparent that the overall status of NK on health and nutrition benefits associated with consumption of FV was dependent on the location of the households. This is clearly illustrated by the fact that the proportion of respondents with good NK was higher among urban than rural respondents by about 11.5%. This implies that urban inhabitants have better NK compared to their rural counterparts. This disparity can be attributed to the fact that more urban respondents attained higher level of education (secondary and above) than their rural counterparts. This is because, whereas nutrition education improves NK [25], the results of this study indicate that the proportion of rural and urban respondents that received nutrition education was somewhat identical. This brings into question the retention of knowledge acquired through nutrition education. It should be appreciated that knowledge accumulation increases with the level of education [21]. Therefore, the higher proportion of urban respondents who were more knowledgeable than rural respondents suggests that urban respondents had retained NK acquired from nutrition education due to higher level of formal education compared to rural respondents whose education levels were generally low.

A positive and significant association observed between NA and FV consumption among urban and rural respondents corroborates with the findings of Okidi et al. [39] reported previously with regard to consumption of wild FV in rural localities of the study area. This suggests that existence of good NA has functional revelance in supporting achievement of RDMIL in the study area. On the other hand, lack of association observed between NK and RDMIL contrasts with the findings of Chung et al. [6] which showed that NK was positively associated with consumption of FV among construction apprentices. This indicates that the level of NK detected in the current study is still insufficient to contribute to fostering achievement of RDMIL.

The results of this study clearly show that whereas NA and NK regarding consumption of FV were good, actual intake was below RDMIL. This suggests that other factors other than NA and NK hindered consumption. A study conducted in City suburb of Paris (France) reported that poverty was one of the factors that hindered some households from consuming FV [5]. However, another study conducted in Chicago (United States of America) revealed that higher income households were more associated with high purchasing and consumption of vegetables as compared to the lower income households [18]. In the current study, application of focus group discussions enabled identification of some of those factors. It was interesting to observe that both rural and urban inhabitants had similar barriers to consumption of FV. The factors were mixed and can generally be grouped into primary agricultural constraints, market limitations, postharvest management limitations, health concerns, social discomfort and policy environment. The significance of agricultural production constraints at the primary level such as pests and diseases, lack of high yielding and drought resistant varieties, and limited access to inputs has largely been studied in relation to crop yields and household income [34]. The current study therefore puts into perspective the relevance of agricultural production constraints to health and nutrition. These results therefore contribute to strengthening the drive towards integration of agriculture into health and nutrition research [4]. The importance of postharvest management limitations relates to the fact that food production in the area, as typical in many parts of the developing world rely on natural weather [30]. As such FV are largely available during production seasons and losses vary from 20% up to 80% [19]. In otherwords, if postharvest management solutions were available in the community and put into use, the lost fractions would be preserved and made available during the off-season periods of the year. Market limitations in terms of distance and prices, especially during off-seasons came out prominently and was more associated with fruits than vegetables. This partly explains the disparity observed between rural and urban respondents in terms of higher consumption of fruits among the latter than recorded for the former respondents.

With regard to health concerns, allergic reactions were the major issue. This has implications on the application of nutrition education to enhance intake of FV in order to confer the expected nutritional and health benefits. This study focused largely on factors that affect `consumption of FV and as such identification of those FV species associated with allergy was outside the scope of this study. Therefore, future studies should identify those plant species so that they can be left out of the promotion efforts. Related to health concerns was the issue of taste dislike for certain FV. Taste preference is one of the factors that affect food choice [7]. Borrowing from the domain of disease management in conventional medicine, patients take drugs not for the preferred taste but as a treatment to acheive good health outcome [54]. Therefore, for such plant species that people donot like the taste, their consumption should be promoted on the basis of health benefits.

Stigma is an important social factor that negatively affects good health-seeking behavior and is well known in the domain of sexually transmitted infections [14]. The current study provides first insight on the negative influence of stigma to healthy food consumption habits. The notion among both rural and urban respondents that consumption of FV is associated with poverty implies that having good NK on health and nutritional benefits associated with consumption of FV may not guarantee that people will adhere to the recommended level of intake because they care more about their social standing. Therefore, future nutrition education targeting consumption of FV should address stigma in the society as a cross-cuting issue. Because of the prevailing policy on wetland protection, respondents viewed it as a barrier to production and consumption of vegetables during the dry season. This is not withstanding the fact that there has to be a delicate balance between environmental intergrity and agricultural production [32]. This finding illustrates lack of knowledge on the need to protect wetlands so as to avert the unpredictable weather patterns currently being experienced globally.

Classically, it is generally expected that good NA and good NK should translate to good nutritional practices. However, a number of previous studies on various aspects of nutrition have demonstrated so and others provided contradictory results leading to a conclusion that good NA and NK donot necessarily lead to good practices [8]. Additionally, whereas multiple regression analysis showed location of the respondent, age of the respondent, education level of the respondent, main source of household income, major occupation of the household head, attendance of health education by the respondent, household size, major souce of household income as predictors of FV consumption among rural and or urban households, they did not come out clearly during FGDs. Therefore in the case of the current study, lack of adherence to the recommended daily intake level for FV (poor practice) despite good NA and NK may largely be attributed to the barriers identified during the FGDs.

## Conclusions

The tennet of this study was hinged on the WHO recommendation that for a healthy living, an adult should achieve RDMIL. To the contrary, the results have demonstrated that in a developing country setting, despite NA and NK associated with consumption of FV, being good, actual intake is generally below RDMIL. By and large, the results suggest that, the inability of inhabitants to translate good NA and NK to the expected consumption practices was largely due to NANK factors including primary production constraints, market limitations, perceived unfavourable environmental policy environment, and social discomfort. A concerted effort is needed to address the NANK factors if adequate intake of fresh FV is to be achieved in developing countries. An important limitation of this study is that the approach of using the person in-charge of food preparation in the household as a respondent may not take into account food items that other members of the household might consume from elsewhere.

## Supplementary Information

**Additional file 1: Supplementary material S1.** Modified questionaire used to collect data on consumption of fruits and vegetables, nutritional knowledge, attitude and socio-dermographic characteriostics of the respondents.

**Additional file 2: Supplementary material S2.** Modified focus group discussion guide for assessing non-attitudinal and non-knowledge based barriers to consumption of fruits and vegetables.

## Data Availability

The datasets used and/or analysed during the current study are available from the corresponding author on request.

## Abbreviations

FAO: Food and Agricultural Organisation of United Nations
FGD: Focus Group Discussion
FV: Fruits and Vegetables
GDLGS: Gulu District Local Government Statistics
NA: Nutritional Attitude
NANK: Non Attitudinal and Non Knowledge
NK: Nutritional Knowlegde
NCDs: Non Communicable Diseases
RMDIL: Recommended Minimum Daily Intake Level
UBOS: Uganda Bureau of Statistics
WHO: World Health Organisation
