# Supplementary material for: Non-attitudinal and non-knowledge based factors constrain households from translating good nutritional knowledge and attitude to achieve the WHO recommended minimum intake level for fruits and vegetables in a developing country setting: evidence from Gulu district, Uganda
Source: BMC Nutr. 2021 Nov 9;7:68. doi: 10.1186/s40795-021-00469-5 (PMC8576922; doi:10.1186/s40795-021-00469-5)
Supplement: Supplementary file 1 — Additional file 1: Supplementary material S1. Modified questionaire used to collect data on consumption of fruits and vegetables, nutritional knowledge, attitude and socio-dermographic characteriostics of the respondents. [file 40795_2021_469_MOESM1_ESM.docx]

**Supplementary material S1: Questionnaire assessing consumption, nutritional knowledge, attitude towards consumption of fruits and vegetables**

**Section 1: Socio-demographic information**

Instructions: Circle the number corresponding to the response that the respondent has given in each case

|  | **QUESTION** | **RESPONSE** | **CHOICE** |
| --- | --- | --- | --- |
| Q1 | Residential area of the respondent | 1=Rural  2=Urban | 1  2 |
| Q2 | Sex of respondent | 1=Male  2=Female | 1  2 |
| Q3 | Age of respondent | ________years |  |
| Q4 | Marital status of the respondent | 1=Single  2=Married  3=Separated  4=Widowed | 1  2  3  4 |
| Q5 | \| Occupation of household head \|  \|  \| \| --- \| --- \| --- \| | 1=Not employed  2=Employed (salaried)  3=Small scale trading  4=Casual labor  5=Farming  6=Any other(specify)   \|  \|  \| \| --- \| --- \| \|  \|  \| | 1  2  3  4  5  6 |
| Q6 | Have you ever attended school | 1=Yes  0=No | 1  0 |
| Q7 | Education level of the respondent and | 1=No formal education  2=Primary  3=Secondary  4=Post-secondary | 1  2  3  4 |
| Q8 | Number of years spent in school (formal education only) | _____________years |  |
| Q9 | Have you ever attended any health education | 1=Yes  2=No | 1  0 |
| Q10 | Numbers of times the respondent has attended health education | 0=Never  1=Once  2=Twice  3=More than twice | 0  1  2  3 |
| Q11 | Have you ever attended any nutrition education | 1=Yes  0=No | 1  0 |
| Q12 | Numbers of times the respondent has attended nutrition education | 0=Never  1=Once  2=Twice  3=More than twice | 0  1  2  3 |
| Q13 | Household size (people who usually eat from the same pot/house) | __________people |  |
| Q14 | What is the main source of your household income | 1=Formal employment  2=Casual labor  3=Small scale business  4=Sale of agriculture produce  5=Farming  6=Any other (specify) | 1  2  3  4  5  6 |
| Q15 | How is food obtained in the household? | 1=Farming  2=Purchase  3=Food aid  4=Transfers from friends  5=Any other (specify | 1  2  3  4  5 |
| Q16 | Who has the primary responsibility of providing food for the household? | 1=Father  2=Mother  3=Both father and mother  4=Relatives  5=Any other (specify) | 1  2  3  4  5 |
| Q17 | Who has the primary responsibility of deciding which type of food is eaten the household? | 1=Husband  2=Wife/mother  3=Both husband and wife  4=Children  5=Any other (specify) | 1  2  3  4  5 |
| Q18 | Who usually decides how family income is used? | 1=Husband  2=Wife  3=both husband and wife  4=Any other (specify) | 1  2  3  4 |

**Section 2. Daily consumption of fruits and vegetables**

1. How many people ate from this household in the last 24 hours? …………………….
2. Did people in this household consume any of the following fruits and vegetables in the past 24 hours?
3. For the fruits and vegetables consumed please estimate the quantity consumed by the household

|  | **Consumed by the household in the last 24 hours (1=Yes, 2=No)** | **Quantity consumed* (g)** | | | |
| --- | --- | --- | --- | --- | --- |
|  |  | **Break fast** | **Snack** | **Lunch** | **Supper** |
| **Vegetables** |  |  |  |  |  |
| Cabbage |  |  |  |  |  |
| Tomatoes |  |  |  |  |  |
| Spinach |  |  |  |  |  |
| Cauliflower |  |  |  |  |  |
| Sweet pepper |  |  |  |  |  |
| Carrot |  |  |  |  |  |
| Amaranthus |  |  |  |  |  |
| Potatoes |  |  |  |  |  |
| Cowpea leaves (boo) |  |  |  |  |  |
| *Hibiscus spp* (malakuang) |  |  |  |  |  |
| Egg plant |  |  |  |  |  |
| *Solanum gilo* (Bitter tomato/Tula) |  |  |  |  |  |
| Vegetables juice |  |  |  |  |  |
| Fresh beans |  |  |  |  |  |
| **Fruits** |  |  |  |  |  |
| Pineapple |  |  |  |  |  |
| watermelon |  |  |  |  |  |
| sweet banana |  |  |  |  |  |
| Avocado |  |  |  |  |  |
| Oranges |  |  |  |  |  |
| Apple |  |  |  |  |  |
| Mangoes |  |  |  |  |  |
| Jackfruit |  |  |  |  |  |
| passionfruit |  |  |  |  |  |
| Fruits juice |  |  |  |  |  |

*Determined by weighing representative portions of the item consumed.

**Section 4. Assessment of nutritional knowledge regarding consumption of fruits and vegetables**

| **Qn. no** | **Questions** | **Responses** | **Scale** |
| --- | --- | --- | --- |
| 1 | Would you tell me the recommended numbers of serving of fruits and vegetables that are supposed to be consumed per day? | 1. Once a day 2. 2-4 times a day 3. 5 times a day | Once =0  5times =1  2-4 times=2 |
| 2 | Consumption of fruits and vegetables is necessary for a healthy living | a) Agree 🞏  b) Disagree 🞏  c) Neither agree nor disagree 🞏  If disagree, why …...  ……………………. | Disagree=0  Agree =1  Neither agree nor disagree =2 |
| 3 | To ensure healthy living, fruits and vegetables should be consumed at least 5 times a day | a) Agree 🞏  b) Disagree 🞏  c) Neither agree nor disagree 🞏  If disagree, why …...  ……………………. | Disagree=0  Agree =1  Neither agree nor disagree =2 |
| 4 | To achieve better health benefits from fruits and vegetables, one should eat a variety of them | a) Agree 🞏  b) Disagree 🞏  c) Neither agree nor disagree 🞏  If disagree, why …...  ……………………. | Disagree=0  Agree =1  Neither agree nor disagree =2 |
| 5 | Fruits and vegetables have no medicinal value | a) Agree 🞏  b) Disagree 🞏  c) Neither agree nor disagree 🞏  If disagree, why …...  ……………………. | Disagree=0  Agree =1  Neither agree nor disagree =2 |
| 6 | Consumption of fruits and vegetables does not help in reducing the risk of getting heart diseases | a) Agree 🞏  b) Disagree 🞏  c) Neither agree nor disagree 🞏  If disagree, why …...  ……………………. | Disagree=0  Agree =1  Neither agree nor disagree =2 |
| 7 | Consuming vegetables reduces the chances of getting diabetes | a) Agree 🞏  b) Disagree 🞏  c) Neither agree nor disagree 🞏  If disagree, why …...  ……………………. | Disagree=0  Agree =1  Neither agree nor disagree =2 |
| 8 | Daily consumption of fruits and vegetables can help in weight management | a) Agree 🞏  b) Disagree 🞏  c) Neither agree nor disagree 🞏  If disagree, why …...  ……………………. | Disagree=0  Agree =1  Neither agree nor disagree =2 |
| 9 | Consumption of fruits and vegetables reduces the risk of getting cancers | a) Agree 🞏  b) Disagree 🞏  c) Neither agree nor disagree 🞏  If disagree, why …...  ……………………. | Disagree=0  Agree =1  Neither agree nor disagree =2 |
| 10 | Fruits and vegetable should be consumed to provide energy to the body | a) Agree 🞏  b) Disagree 🞏  c) Neither agree nor disagree 🞏  If disagree, why …...  ……………………. | Disagree=0  Agree =1  Neither agree nor disagree =2 |
| 11 | Fruits and vegetables are necessary for body building (protein source) | a) Agree 🞏  b) Disagree 🞏  c) Neither agree nor disagree 🞏  If disagree, why …...  ……………………. | Disagree=0  Agree =1  Neither agree nor disagree =2 |
| 12 | Fruits and vegetables protect me against diseases | a) Agree 🞏  b) Disagree 🞏  c) Neither agree nor disagree 🞏  If disagree, why …...  ……………………. | Disagree=0  Agree =1  Neither agree nor disagree =2 |
| 13 | Fruits and vegetables should be washed after cutting | a) Agree 🞏  b) Disagree 🞏  c) Neither agree nor disagree 🞏  If disagree, why …...  ……………………. | Disagree=0  Agree =1  Neither agree nor disagree =2 |
| 14 | Fruits and vegetables can be a source of some diseases | a) Agree 🞏  b) Disagree 🞏  c) Neither agree nor disagree 🞏  If disagree, why …...  ……………………. | Disagree=0  Agree =1  Neither agree nor disagree =2 |
| 15 | Overcooking fruits and vegetables reduce their nutritional value | a) Agree 🞏  b) Disagree 🞏  c) Neither agree nor disagree 🞏  If disagree, why …...  ……………………. | Disagree=0  Agree =1  Neither agree nor disagree =2 |
| 16 | Fruits and vegetables such as tomatoes, onions, carrots, and green pepper should largely be consumed in a raw form | a) Agree 🞏  b) Disagree 🞏  c) Neither agree nor disagree 🞏  If disagree, why …...  ……………………. | Disagree=0  Agree =1  Neither agree nor disagree =2 |

**Section 4. Assessment of attitude toward consumption of fruits and vegetables**

| **Q. no** | **Questions** | **Responses** | **Scale** |
| --- | --- | --- | --- |
| 1 | I believe fruits and vegetables should always be included in my diet | a) Agree 🞏  b) Disagree 🞏  c) Neither agree nor disagree 🞏  If disagree, why …...  ……………………. | Disagree=0  Agree =1  Neither agree nor disagree =2 |
| 2 | I do believe a balanced diet should always have fruits and vegetables | a) Agree 🞏  b) Disagree 🞏  c) Neither agree nor disagree 🞏  If disagree, why …...  ……………………. | Disagree=0  Agree =1  Neither agree nor disagree =2 |
| 3 | I do believe consumption of fruits and vegetables can help in weight management | a) Agree 🞏  b) Disagree 🞏  c) Neither agree nor disagree 🞏  If disagree, why …...  ……………………. | Disagree=0  Agree =1  Neither agree nor disagree =2 |
| 4 | I consume fruits and vegetables because they are a good source of vitamins | a) Agree 🞏  b) Disagree 🞏  c) Neither agree nor disagree 🞏  If disagree, why …...  ……………………. | Disagree=0  Agree =1  Neither agree nor disagree =2 |
| 5 | I only consume fruits and vegetables when they are available | a) Agree 🞏  b) Disagree 🞏  c) Neither agree nor disagree 🞏  If disagree, why …...  ……………………. | Disagree=0  Agree =1  Neither agree nor disagree =2 |
| 6 | I ensure that fruits and vegetables are included in my diet every day | a) Agree 🞏  b) Disagree 🞏  c) Neither agree nor disagree 🞏  If disagree, why …...  ……………………. | Disagree=0  Agree =1  Neither agree nor disagree =2 |
| 7 | Even if fruits and vegetables are consumed in my family or not, I am not concerned | a) Agree 🞏  b) Disagree 🞏  c) Neither agree nor disagree 🞏  If disagree, why …...  ……………………. | Disagree=0  Agree =1  Neither agree nor disagree =2 |
| 8 | I don’t believe it is always necessary to clean fruits and vegetables before consumption | a) Agree 🞏  b) Disagree 🞏  c) Neither agree nor disagree 🞏  If disagree, why …...  ……………………. | Disagree=0  Agree =1  Neither agree nor disagree =2 |
| 9 | I consider it important to know the source of fruits and vegetables before buying them | a) Agree 🞏  b) Disagree 🞏  c) Neither agree nor disagree 🞏  If disagree, why …...  ……………………. | Disagree=0  Agree =1  Neither agree nor disagree =2 |
| 10 | It is important to know where the fruits and vegetables are grown before consumption | a) Agree 🞏  b) Disagree 🞏  c) Neither agree nor disagree 🞏  If disagree, why …...  ……………………. | Disagree=0  Agree =1  Neither agree nor disagree =2 |
| 11 | As long as I take other food types, I do not find it necessary to eat fruits and vegetables | a) Agree 🞏  b) Disagree 🞏  c) Neither agree nor disagree 🞏  If disagree, why …...  ……………………. | Disagree=0  Agree =1  Neither agree nor disagree =2 |
| 12 | I believe I can still be healthy without consuming fruits and vegetables | a) Agree 🞏  b) Disagree 🞏  c) Neither agree nor disagree 🞏  If disagree, why …...  ……………………. | Disagree=0  Agree =1  Neither agree nor disagree =2 |
| 13 | I care about my safety as I consume fruits and vegetables | a) Agree 🞏  b) Disagree 🞏  c) Neither agree nor disagree 🞏  If disagree, why …...  ……………………. | Disagree=0  Agree =1  Neither agree nor disagree =2 |
| 14 | I believe it is safer to consume only locally produced fruits and vegetables | a) Agree 🞏  b) Disagree 🞏  c) Neither agree nor disagree 🞏  If disagree, why …...  ……………………. | Disagree=0  Agree =1  Neither agree nor disagree =2 |
| 15 | I believe fruits and vegetables from rural areas are safer than those from urban areas | a) Agree 🞏  b) Disagree 🞏  c) Neither agree nor disagree 🞏  If disagree, why …...  ……………………. | Disagree=0  Agree =1  Neither agree nor disagree =2 |
| 16 | I buy fruits and vegetables because they are cheaper than other foods | a) Agree 🞏  b) Disagree 🞏  c) Neither agree nor disagree 🞏  If disagree, why …...  ……………………. | Disagree=0  Agree =1  Neither agree nor disagree =2 |
| 17 | I believe that fruits and vegetables should be consumed by the poor | a) Agree 🞏  b) Disagree 🞏  c) Neither agree nor disagree 🞏  If disagree, why …...  ……………………. | Disagree=0  Agree =1  Neither agree nor disagree =2 |
| 18 | I think I can easily find any kind of fruit & vegetable in my locality | a) Agree 🞏  b) Disagree 🞏  c) Neither agree nor disagree 🞏  If disagree, why …...  ……………………. | Disagree=0  Agree =1  Neither agree nor disagree =2 |
| 19 | I believe home grown fruits and vegetables are safer that those sold in the market | a) Agree 🞏  b) Disagree 🞏  c) Neither agree nor disagree 🞏  If disagree, why …...  ……………………. | Disagree=0  Agree =1  Neither agree nor disagree =2 |
| 20 | I find eating fruits and vegetables multiple times daily burdensome | a) Agree 🞏  b) Disagree 🞏  c) Neither agree nor disagree 🞏  If disagree, why …...  ……………………. | Disagree=0  Agree =1  Neither agree nor disagree =2 |
| 21 | I anticipate fruits and vegetables not to be nutritious | a) Agree 🞏  b) Disagree 🞏  c) Neither agree nor disagree 🞏  If disagree, why …...  ……………………. | Disagree=0  Agree =1  Neither agree nor disagree =2 |
| 22 | I anticipate fruits and vegetables to be unsafe | a) Agree 🞏  b) Disagree 🞏  c) Neither agree nor disagree 🞏  If disagree, why …...  ……………………. | Disagree=0  Agree =1  Neither agree nor disagree =2 |
| 23 | I anticipate fruits and vegetables consumption to be demeaning | a) Agree 🞏  b) Disagree 🞏  c) Neither agree nor disagree 🞏  If disagree, why …...  ……………………. | Disagree=0  Agree =1  Neither agree nor disagree =2 |
| 24 | I would prefer fruits and vegetable consumption during periods of food scarcity | a) Agree 🞏  b) Disagree 🞏  c) Neither agree nor disagree 🞏  If disagree, why …...  ……………………. | Disagree=0  Agree =1  Neither agree nor disagree =2 |
| 25 | I should only eat fruits and vegetables when I feel like | a) Agree 🞏  b) Disagree 🞏  c) Neither agree nor disagree 🞏  If disagree, why …...  ……………………. | Disagree=0  Agree =1  Neither agree nor disagree =2 |
| 26 | Regular consumption of fruits and vegetables is a sign of poverty | a) Agree 🞏  b) Disagree 🞏  c) Neither agree nor disagree 🞏  If disagree, why …...  ……………………. | Disagree=0  Agree =1  Neither agree nor disagree =2 |
| 27 | I would only consume fruits and vegetables if recommended by the Medical doctor or personnel | a) Agree 🞏  b) Disagree 🞏  c) Neither agree nor disagree 🞏  If disagree, why …...  ……………………. | Disagree=0  Agree =1  Neither agree nor disagree =2 |
| 28 | I find washing fruits and vegetables before consumption burdensome | a) Agree 🞏  b) Disagree 🞏  c) Neither agree nor disagree 🞏  If disagree, why …...  ……………………. | Disagree=0  Agree =1  Neither agree nor disagree =2 |
