# Supplementary material for: Non-attitudinal and non-knowledge based factors constrain households from translating good nutritional knowledge and attitude to achieve the WHO recommended minimum intake level for fruits and vegetables in a developing country setting: evidence from Gulu district, Uganda
Source: BMC Nutr. 2021 Nov 9;7:68. doi: 10.1186/s40795-021-00469-5 (PMC8576922; doi:10.1186/s40795-021-00469-5)
Supplement: Supplementary file 2 — Additional file 2: Supplementary material S2. Modified focus group discussion guide for assessing non-attitudinal and non-knowledge based barriers to consumption of fruits and vegetables. [file 40795_2021_469_MOESM2_ESM.docx]

**Supplementary material S2: Focus group discussion guide for assessing non-attitudinal and non-knowledge-based barriers to consumption of fruits and vegetables**

**Guiding questions**

**Preamble:** High of level of incidences of mortality attributed to non-communicable diseases such as cancer, diabetes and hypertension are being experienced in Uganda. The World Health Organization (WHO) recommends that people should consume adequate amounts of fruits and vegetables (at least 400g/five (5) servings per day per person), so as to reduce the risk of development of such diseases. Household assessment conducted a few weeks ago showed that people in Gulu district do not meet the recommended minimum daily level of consumption of fruits and vegetables.

We would like to discuss with you the reasons as to why the households are not meeting the recommendation:

1. In this community, what factors discourage/constrain people from achieving the recommended minimum daily intake of fruits and vegetables?
2. How does each of the factors mentioned above discourage/constrain people from achieving adequate consumption of fruits and vegetables?
3. Are there any other concerns with respect to inadequate consumption of fruits and vegetables in this community? Please explain

Thank you for your participation
